# Short-Term High-Altitude Exposure Alters Pharyngeal and Gut Microbiome

**DOI:** 10.1021/envhealth.5c00585

**Published:** 2026-01-14

**Authors:** Yi Zhang, Xin Meng, Jicheng Gong, Yifan Xu, Qiaoyi Hua, Wu Chen, Teng Wang, Xudong He, Ruiwei Xu, Yingfeng Gao, Chunxiang Ye, Cencen Wu, Yuanyuan Fan, Lingyun Zu, Tong Zhu

**Affiliations:** 1 MEEKL-AERM, College of Environmental Sciences and Engineering, Institute of Tibetan Plateau, and Center for Environment and Health, 12465 Peking University, Beijing 100871, China; 2 Institute of Tibetan Plateau, 12465 Peking University, Beijing 100871, China; 3 TUWAS-AEEEH, Xizang University, Lhasa, Xizang 850000, China; 4 Medical College of Xizang University, Lhasa, Xizang 850000, China; 5 Department of Cardiology and Institute of Vascular Medicine, Peking University Third Hospital, State Key Laboratory of Vascular Homeostasis and Remodeling, Peking University, NHC Key Laboratory of Cardiovascular Molecular Biology and Regulatory Peptides, Peking University, Beijing Key Laboratory of Cardiovascular Receptors Research, Beijing 100191, China

**Keywords:** high-altitude exposure, pharyngeal microbiome, gut microbiome, acclimatization, microbial diversity

## Abstract

High-altitude exposure poses significant health challenges, where microbial communities may serve as important contributors to host acclimatization. We conducted a longitudinal study to investigate the dynamic changes in pharyngeal and gut microbiota before, during, and after acclimatization to the Mount Qomolangma region (high-altitude, 4300–5200 m above sea level, m.a.s.l.). Twenty healthy participants underwent four health visits: at baseline level (Beijing, 50 m.a.s.l.), upon arrival at the high altitude, after a week of acclimatization, and upon return to the baseline level. Throat swabs and fecal samples were collected for 16S rRNA amplicon sequencing to assess microbial composition and diversity. Linear mixed-effects models were employed to estimate the altitude-associated variations in pharyngeal and gut microbes compared to the baseline. Pharyngeal microbial diversity showed statistically significant alterations after 1 week at high altitude, with the Shannon index decreasing by 12.0% (95% CI: −21.8 to −2.9%) and the Simpson index increasing by 2.6% (1.3 to 4.0%). In contrast, gut microbial diversity decreased upon initial high-altitude exposure but tended to revert to baseline after 1 week of acclimatization. Beta diversity analyses revealed significant differences in pharyngeal microbiota across visits, while gut microbiota differences were less pronounced. Using the linear discriminant analysis effect size (LEfSe) method, we identified 11 pharyngeal and four gut microbes that were differentially abundant across visits, which could shape the host’s resilience to high-altitude challenges. Our study reveals that high-altitude exposure disrupts pharyngeal and gut microbial diversity over time and modulates the abundance of some opportunistic pathogens. These shifts may mediate host responses to hypoxic environments, underscoring the microbiomes’ role in physiological acclimatization.

## Graphical abstract

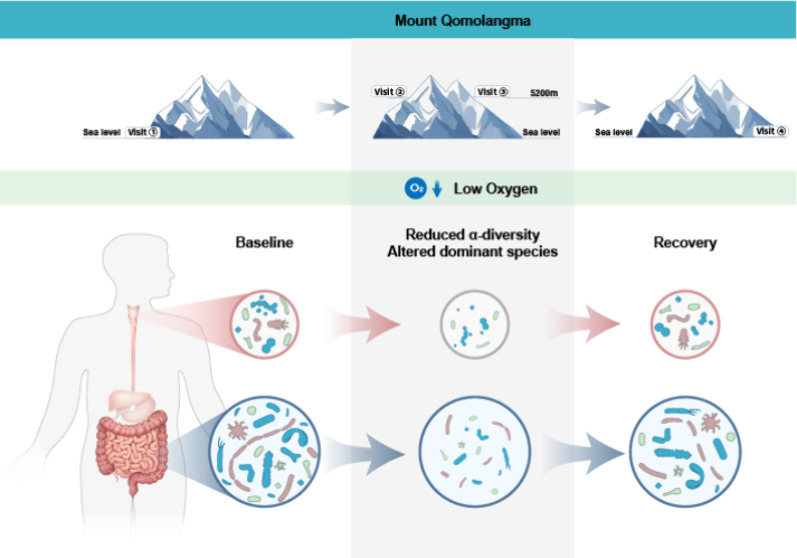

## Introduction

High-altitude environments, characterized by hypoxia, pose significant physiological challenges to humans.^1^ With over 100 million people visiting high-altitude regions annually,^2^ rapid transitions often trigger acute altitude sickness, including headaches, dyspnea, and nausea.^3^ Beyond traditional physiological mechanisms, the human microbiome is increasingly recognized as a critical factor in high-altitude acclimatization.^4^ The symbiotic microbiota, known to influence systemic physiology and immune responses,^5,6^ exhibit dynamic changes during altitude exposure, suggesting their involvement in high-altitude acclimatization.^7^

The human microbiome is highly sensitive to environmental changes and is crucial for health and physiological regulation.^8^ Research shows that high-altitude residents exhibit unique microbial diversity and community composition compared to lowland populations, enhancing their adaptability to extreme environments.^9–11^ However, microbiome responses in lowlanders who acutely ascend to high altitudes remain understudied.^12^ Understanding microbiome changes during short-term exposure could provide insights into preventing altitude-related illnesses and developing interventions to enhance acclimatization, benefiting the growing population of high-altitude sojourners.

Considering the pronounced individual variability of the microbiome, longitudinal studies tracking microbial changes at individual levels offer a practical approach to understanding these dynamics during high-altitude acclimatization. While evidence suggests that gut microbiota may facilitate acclimatization to hypoxia by modulating anti-inflammatory mechanisms and intestinal barrier protection,^12,13^ the broader role of microbiome in physiological responses to high-altitude exposure remains unclear. Moreover, research on microbiome dynamics in nongut sites during acclimatization, particularly in the upper respiratory tract, remains limited, despite their potential involvement in host-environment interactions at high altitudes.

To address these research gaps, we conducted a longitudinal study in subjects who rapidly ascended to the Mount Qomolangma region (4300 to 5200 m above sea level, m.a.s.l.), to examine pharyngeal and gut microbial responses before, during, and after the high-altitude exposure. Throat swabs and fecal samples were collected and analyzed using 16S rRNA amplicon sequencing to investigate the composition and structure of the pharyngeal and gut microbiota. These microbial shifts are hypothesized to play a critical role in the host’s physiological acclimatization to high-altitude conditions.

## Methods

### Study Design and Participants

We employed a quasi-experimental design to study changes in pharyngeal and gut microbiota among 20 healthy lowlanders during a scientific expedition from the baseline at sea level (Beijing, 50 m.a.s.l.) to high altitude (4300–5200 m.a.s.l.). Eligible participants were 18–60 years old, had no history of cardiovascular, respiratory, digestive, or allergic disease, were not receiving chronic pharmacological treatment, had not experienced severe high-altitude illness, and had not been exposed to altitudes above 2500 m a.s.l. in the preceding two months; all underwent four health assessments. Baseline measurements (Visit 1) were taken in Beijing, and in a week, participants traveled to the high altitude of the Mount Qomolangma region, with the second visit (Visit 2) completed within 5 days of arrival. After a seven-day acclimatization period, the third visit (Visit 3) was conducted at the Qomolangma region. Participants returned to Beijing after a two-week stay at the Qomolangma region and completed the final visit (Visit 4) within two months (three and a half months for two participants). Detailed study design can be found in the previous research.^14^

All health examinations were conducted in the morning (8:00–9:30 AM, Beijing Time), and participants were required to fast for at least 8 h prior to each visit. Throat swabs and fecal samples were collected from fasting participants and were immediately stored at – 80 °C (Visits 1 and 4) or on dry ice (Visits 2 and 3) until further analysis. At each visit, a brief questionnaire was administered to obtain information on oxygen supplementation, antibiotic use, and symptoms experienced during the 3 days preceding sampling. Peripheral capillary oxygen saturation (SpO_2_) was noninvasively assessed using an O_2_ Ring finger-probe oximeter (Lepu Medical Technology, China), and the mean SpO_2_ during the visit was calculated for each participant. Fasting venous blood samples were collected and transported to Dingri County People’s Hospital for routine hematological testing, from which hemoglobin (HGB) concentrations were obtained.

The study was approved by the Institutional Review Board of Peking University Health Science Center (IRB00001052–19062), and all participants provided written informed consent.

### Microbiota Analysis

All 79 throat swab samples and 71 fecal samples were subjected to genomic DNA extraction and a high-quality Illumina sequencing process. Pharyngeal microbial DNA was extracted from throat swab samples using the FastDNA SPIN Kit for Soil (MP Biomedicals, Santa Ana, CA, USA), whereas gut microbial DNA from fecal samples was extracted using the E.Z.N.A. Soil DNA Kit (Omega Biotek, Norcross, GA, USA). We used the 16S rRNA gene with the V3–V4 highly variable region of the bacteria. The V3–V4 hypervariable regions of the bacterial 16S rRNA gene were amplified using primers 338 F (50-ACTCCTACGGGAGGCAGCAG-30) and 806 R (50-GGACTACHVGGGTWTCTAAT-30) by a thermocycler PCR system (GeneAmp 9700, ABI, Waltham, MA, USA). The resulting PCR products were extracted from a 2% agarose gel and further purified using the AxyPrep DNA Gel Extraction Kit (Axygen Biosciences, Union City, CA, USA) and quantified using QuantiFluor-ST (Promega, Madison, WI, USA) according to the manufacturer’s protocol.

Purified amplicons were pooled in equimolar quantities and paired-end sequenced (2 × 300) on an Illumina MiSeq platform (Illumina, San Diego, CA, USA) according to the standard protocols by Majorbio Bio-Pharm Technology Co. Ltd. (Shanghai, China). Raw 16S rRNA sequencing data were demultiplexed, quality-filtered by Trimmomatic, and merged using the FLASH technique and processed in QIIME2 (v2022.2). The DADA2 plugin was used for quality control and to identify amplicon sequence variants (ASVs), representative sequences, and abundance information. The taxonomy of each 16S rRNA gene sequence was assigned using the classify-sklearn (Naive Bayes) algorithm implemented in QIIME2 (v2022.2), with the Silva reference database (v.138) and a confidence threshold of 70%. Before calculating diversity indices, the ASV count table was normalized using total sum scaling (TSS) and rarefied to the minimum sequencing depth across samples to account for differences in library size.

### Statistical Analysis

To analyze microbial community changes under high-altitude exposure, we employed a comprehensive statistical approach. Alpha-diversity indices, including Shannon, Simpson, Chao 1, abundance-based coverage estimator (ACE), and observed species (Sobs), were calculated using Mothur (Version 1.30.2) to evaluate species richness and evenness. Beta diversity was assessed using the Bray–Curtis distance matrix derived from genus-level data, followed by principal coordinate analysis (PCoA) to visualize compositional differences among samples. To quantify the impact of altitude variation, permutational multivariate analysis of variance (PERMANOVA) was performed on the Bray–Curtis distance matrix across the four study visits.

To identify the distinguishing taxa at the genus level, the linear discriminant analysis (LDA) effect size (LEfSe) method was implemented (https://www.microbiomeanalyst.ca/). Taxa were considered differential microbes if they had an LDA score >2 and *p* < 0.05 in the LEfSe analysis, and these were included in subsequent correlation analyses. Linear mixed-effects (LME) models were applied to assess intraindividual variability in repeated measurements. Alpha diversity indexes and the relative abundance of microorganisms at the genus level served as response variables that were transformed by natural logarithms before the model construction. The effects of high-altitude exposure were examined using four health visits as a categorical variable to reflect altitude changes. The models were adjusted for age, gender, smoking status, body mass index (BMI), and antibiotic use. Subject ID was treated as a random intercept. All estimates are reported as percentage differences with 95% confidence intervals (CIs) relative to the baseline visit. These analyses were conducted using the “lme4” package in R software (version 4.4.1). The covariation between differential microbial genera and hypoxia-related physiological indices was assessed using Spearman’s rank correlation analysis.

Statistical significance was defined as two-sided *p* values <0.05, with adjustment for multiple comparisons using the Benjamini–Hochberg method, restricting an overall false discovery rate (FDR) of 5%.

## Results

### Demographic Characteristics

The characteristics of the study participants are summarized in Table 1. After excluding one participant due to the missing data at baseline, we included 20 participants (12 males and eight females) with 79 health visits in the data analysis. During these visits, 79 throat swabs (100%) and 71 fecal samples (89%) were collected. The participants had an average age of 32.3 years old and a mean BMI of 22.8 kg/m^2^. Most participants were nonsmokers (80%), and all had at least a university degree. In the high-altitude region, 55% of participants reported gastrointestinal symptoms, while 10% experienced respiratory symptoms. Additionally, 50% of the participants used oxygen supplementation, and 10% received antibiotics during their high-altitude exposure. However, oxygen use was sporadic, brief, and noncontinuous, predominantly in the form of canned oxygen or diffuse oxygen devices, with a total cumulative oxygen exposure of no more than 3 h per participant over the entire campaign. Intermittent oxygen use reflects routine symptomatic management during high-altitude sojourns and does not indicate removal from the hypoxic environment. Across all visits, only two antibiotic-exposed person-visits were recorded, occurring 6 days and 10 days before sampling, respectively. Detailed information on oxygen supplementation and antibiotic use is provided in Table S1 and Table S2.

**Table 1:** Characteristics of the Study Participants

	*N* = 20^a^
demographic characteristics	
age, year	32.3 ± 10.8
body mass index, kg/m^2^	22.8 ± 2.7
sex	
male	12 (60)
female	8 (40)
smoking status	
never smokers	16 (80)
current smokers	4 (20)
education level	
university	20 (100)
below university	0 (0)
birthplace altitude (m)	
<1500	16 (80)
1500–2500	4 (20)
symptoms at high altitude	
gastrointestinal symptoms	
absent	9 (45)
presence	11(55)
respiratory symptoms	
absent	18 (90)
presence	2 (10)
altitude sickness treatment	
oxygen supplementation	
yes	10 (50)
no	10 (50)
antibiotic use	
yes	2 (10)
no	18 (80)

a Data are shown in numbers (%) or means ± standard deviations.

### Microbiota Diversities during the Altitude Acclimatization

A total of 13,414,625 sequences were obtained from participants’ throat swabs, and 10,407,513 sequences were collected from fecal samples, with average lengths of the sequences being 413 bp and 421 bp, respectively. After preprocessing the sequences, the pharyngeal microbiota comprised seven phyla, 28 families, and 39 genera, while the gut microbiota revealed nine phyla, 48 families, and 114 genera. We calculated both the alpha and beta diversity of microbial communities at the taxonomic genus level, with statistics for alpha diversity in pharyngeal and gut microbiota reported in Table S3.

LME models showed that HGB levels increased significantly, whereas SpO_2_ decreased significantly during high-altitude exposure (Figure S1), indicating that participants were in a pronounced hypoxic state throughout the high-altitude stay.

Figure 1 presents changes in alpha diversity indexes during the high-altitude acclimatization. The pharyngeal microbiota showed a delayed temporal pattern, with changes emerging after 1 week of acclimation, where the Shannon index decreased by 12% (95% CI: −21.8% to −2.9%) and the Simpson index increased by 2.6% (95% CI: 1.3% to 4.0%). In contrast, for the gut microbiota, the Shannon index decreased and the Simpson index increased immediately upon initial high-altitude exposure, both indicating reduced microbial diversity, with such changes tending to return to baseline levels after a week of acclimatization. We did not observe convincing changes in indices concerning ACE, Chao1, and Sobs for either pharyngeal or gut microorganisms.

**Figure 1:**
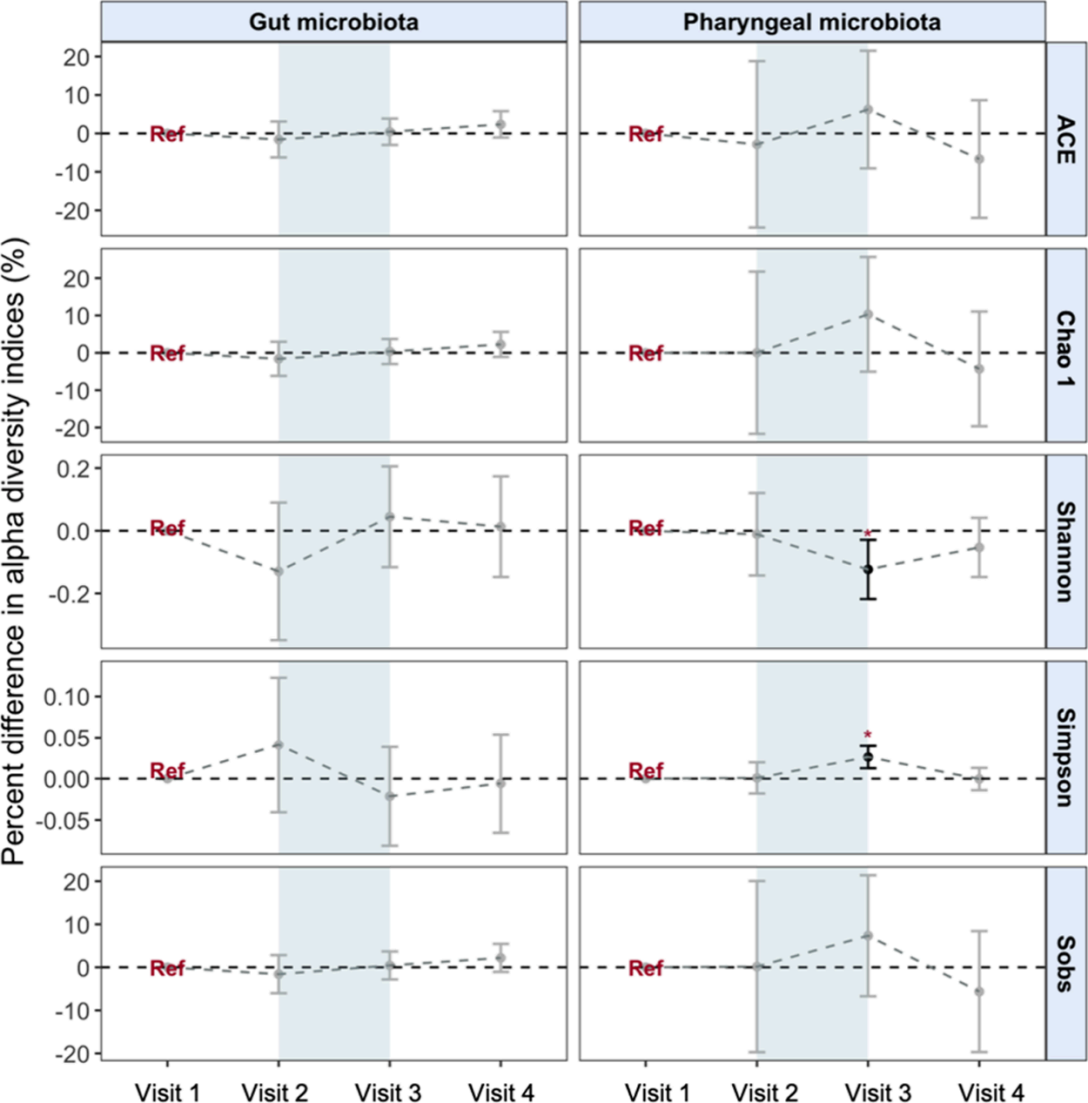
Differences in alpha diversity indices of gut and pharyngeal microbiota at four visits. Results were obtained from linear mixed-effects models with random intercepts of participants and adjusted for age, gender, smoking status, body mass index (BMI), and antibiotic use. All estimates are reported as percent differences with 95% confidence intervals compared with the baseline (visit 1), and no intersection with the horizontal dotted line indicates significant differences (in black). Differences with FDR_B–H_ < 0.05 are marked with asterisks. Blue shading indicates visits conducted at the high-altitude region.

Regarding beta diversity, PCoA results suggest that pharyngeal microbiota showed significant differences across four visits on the genus level generated based on the Bray–Curtis Index (R^2^ = 0.097, *p* = 0.002) (Figure S2). Results of pairwise PERMANOVA show that all other pairwise comparisons between visits reached statistical significance, except for the two measurements at sea level, whose FDR is 0.358 (Table S4). Although the results of pairwise PERMANOVA and PCoA in gut microbiota are also not statistically significant, they still suggest that the difference between the stay at high altitude (Visit 2 and Visit 3) and the sea level (Visit 1 and Visit 4) is higher than between the high-altitude group (Visit 2 vs Visit 3) and between the sea-level group (Visit 1 vs Visit 4). Of the four visits, Visit 1 and Visit 3 showed the greatest differences. With prolonged plateau exposure, both pharyngeal and gut microbial composition appeared to show greater differences compared to baseline levels.

### Specific Species Changes with Altitude

Figure 2 and Figure 3 show the intergroup differences in pharyngeal and gut microbiota at the family and genus levels, respectively. We employed the LEfSe method to identify the distinguishing taxa at the genus level across four visits, with criteria of LDA > 2 and *p* < 0.05. A total of 11 pharyngeal and 4 gut microorganisms were selected under this criterion, and we further associated these microbial genera with altitude groupings. The results of the LEfSe analysis are shown in Table S5 and Table S6, while the relative abundance of the differential pharyngeal and gut microbiota across four visits is presented in Figure S3.

**Figure 2:**
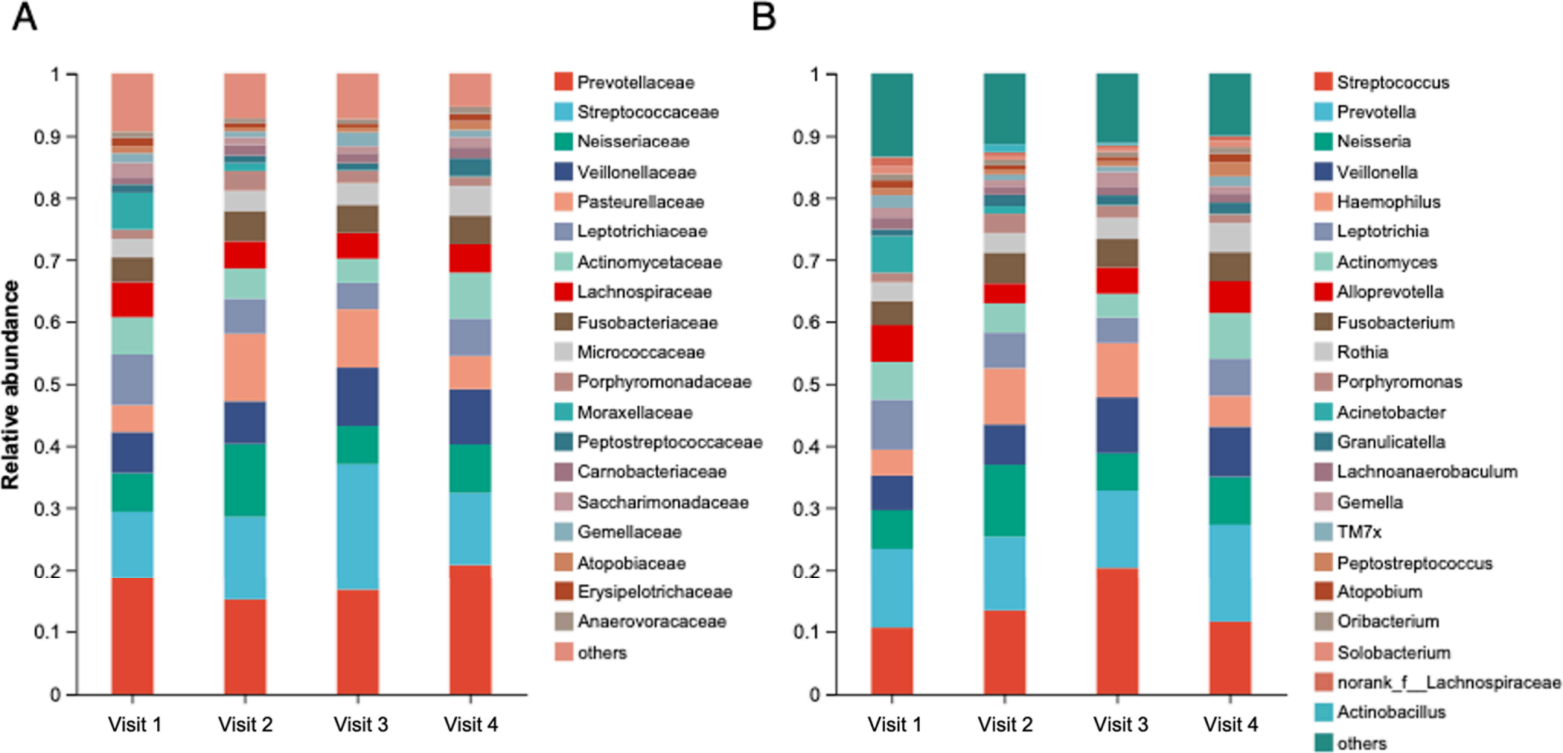
Intergroup differences of pharyngeal microbiota at family level (A) and genus level (B). Species with a relative abundance of >1% per sample are plotted.

**Figure 3:**
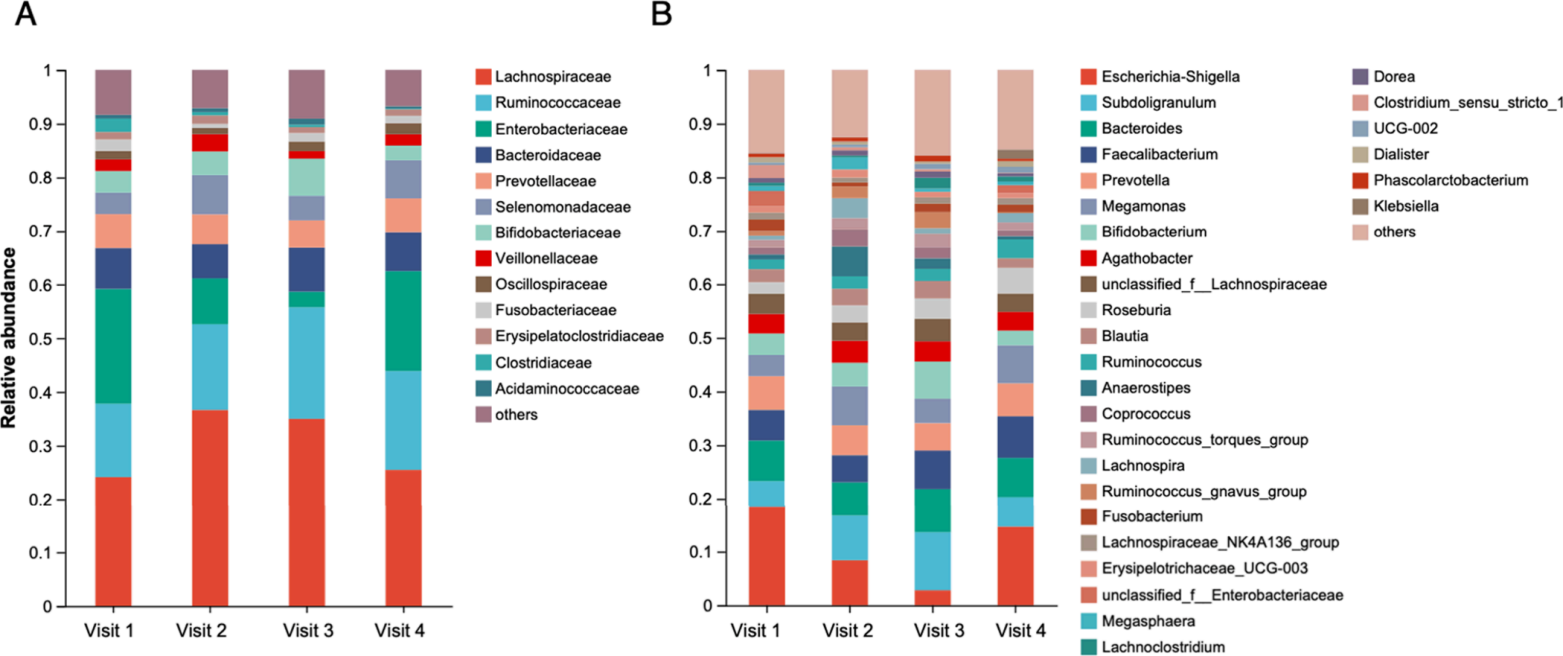
Intergroup differences of gut microbiota at family level (A) and genus level (B). Species with a relative abundance of >1% per sample are plotted.

As shown in Figure 4, high-altitude exposure induced significant shifts in pharyngeal microbiota. During high-altitude exposure, *Haemophilus*, *Porphyromonas*, *Streptococcus*, and *f_Actinomycetaceae_g_F0332* showed significant elevations of 5.12% (95%CI: 2.81% to 7.44%), 1.57% (95%CI: 0.74% to 2.39%), 10.20 (95%CI: 5.79% to 14.60%), and 0.07% (95%CI: 0.03% to 0.12%), respectively. *Lachnoclostridium* and *Novosphingobium* showed a marginally significant elevated trend during high-altitude exposures (*p* < 0.1). *Solobacterium* displayed progressive reduction during high-altitude exposure, with a maximum decrease of −0.76% (95%CI: −1.13% to −0.39%), and showed recovery after returning to the baseline level. Both *Alloprevotella* and *Atopobium* showed significant decreases at the onset of high-altitude exposure, declining by 2.86% (95%CI: −5.32% to −0.40%) and 0.56% (95%CI: −1.08% to −0.04%), respectively. *Bergeyella* tended to increase during high-altitude exposure, while *Delftia* and *Acinetobacter* showed a tendency to decrease, although these changes were not statistically significant. *Peptostreptococcus* did not exhibit significant changes during high-altitude exposure, but it increased significantly by 0.99% (95% CI: 0.01% to 1.98%) upon returning to sea level.

**Figure 4:**
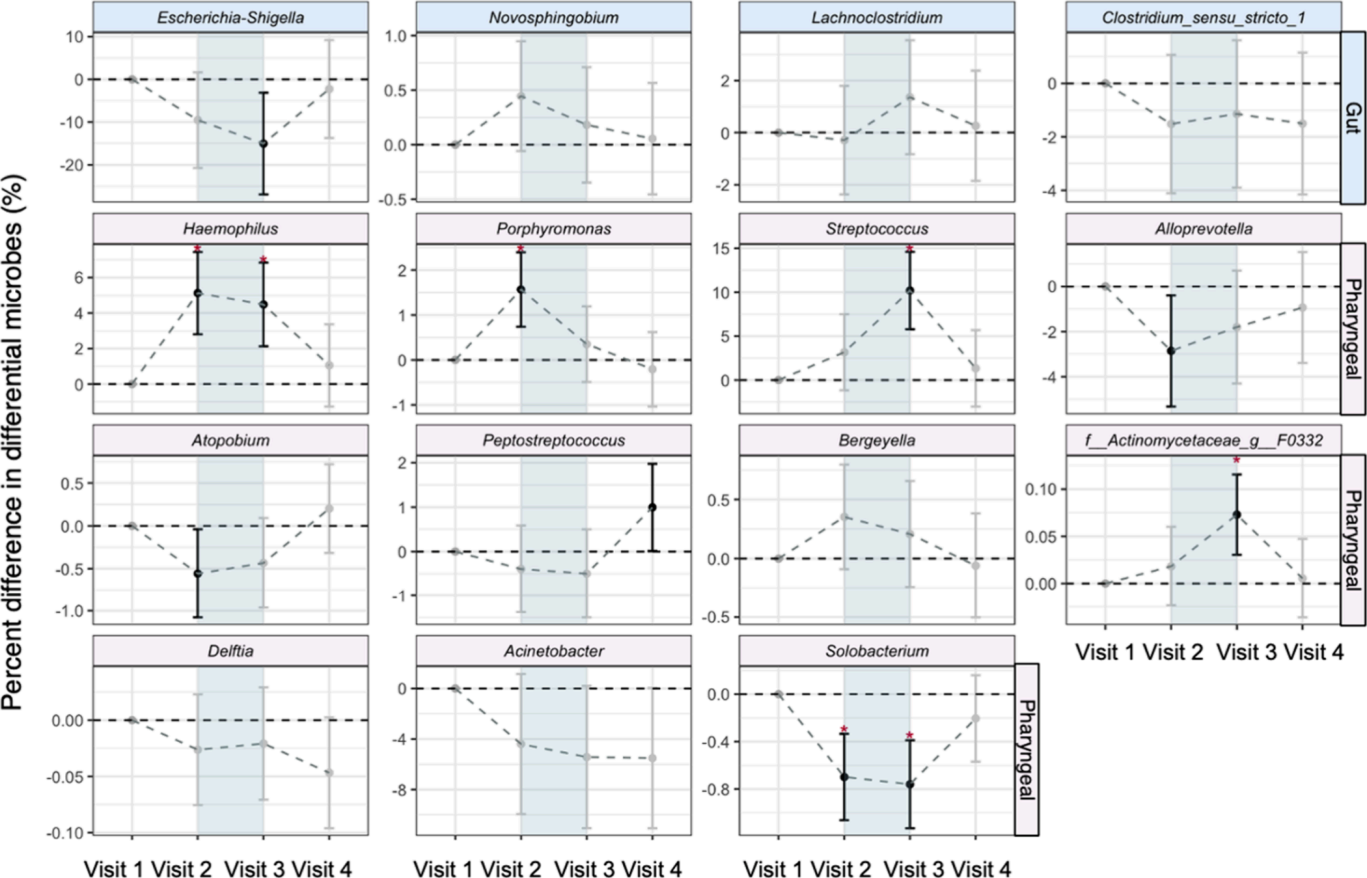
Differences in the differential gut and pharyngeal microbiota at four visits. Results were obtained from linear mixed-effects models with random intercepts of participants and adjusted for age, gender, smoking status, body mass index (BMI), and antibiotic use. All estimates are reported as percent differences with 95% confidence intervals compared with the baseline visit (visit 1), and no intersection with the horizontal black dotted line indicates significant differences (in black). Differences with FDR_B–H_ < 0.05 are marked with asterisks. Blue shading indicates visits conducted at the high-altitude region.

As for the gut microbe, we found that *Escherichia-Shigella* showed a decreasing trend right after the initial entry into the high-altitude area and showed a significant decrease by 15.03% (95%CI: −26.92% to −3.14%) after a period of continuous exposure (Visit 3), but was able to return to the baseline level after returning to the sea level (Visit 4).

We examined the distribution of the aforementioned microbes after categorizing the subjects based on the absence or presence of gastrointestinal or respiratory symptoms (Figure S4). Relative enrichment of *Acinetobacter*, *Delftia*, and *Bergeyella* in the pharynx was found in subjects presenting respiratory symptoms. Among subjects who self-reported gastrointestinal symptoms, the relative abundance of *Escherichia-Shigella* and *Lachnoclostridium* was found to be lower, while that of *Novosphingobium* was higher.

Spearman rank correlations between differential pharyngeal and gut genera and hypoxia-related physiological indices, HGB and SpO_2_, are shown in Figure S5. The four gut genera that differed by altitude all exhibited concordant positive correlations with HGB and concordant inverse correlations with SpO_2_, although none of these associations reached statistical significance (all *p* > 0.05). In contrast, most pharyngeal genera showed the same correlations with HGB and SpO_2_ in the positive direction, except for *Delftia* and *f_Actinomycetaceae_g_F0332*, which displayed discordant patterns.

### Sensitivity Analyses

In sensitivity analyses excluding the two visits with antibiotic use prior to clinical sampling, the associations between altitude-related visit phases and both alpha-diversity indices and specific pharyngeal and gut genera remained robust, with effect estimates and statistical significance largely unchanged (Figure S6, Figure S8). When oxygen supplementation was further included as a covariate, the directions and magnitudes of the associations also remained broadly consistent, although the statistical significance for some individual taxa was slightly attenuated or strengthened (Figure S7, Figure S9). Specifically, the decrease in *Escherichia–Shigella* at Visit 2, the increase in *Novosphingobium* at Visit 2, and the decrease in *Acinetobacter* at Visit 3 became statistically significant, whereas the reductions in *Alloprevotella* and *Atopobium* at Visit 2 were no longer statistically significant. Overall, the main patterns of altitude-related changes in pharyngeal and gut microbiota remained qualitatively robust after excluding antibiotic-exposed visits and additionally adjusting for oxygen supplementation.

## Discussion

This longitudinal study examined the dynamic changes in pharyngeal and gut microbiota before, during, and after a high-altitude acclimatization. We found that both microbiomes experienced diversity reductions but followed different temporal patterns. Notably, pharyngeal microbiota showed significant structural shifts, whereas gut microbiota showed greater stability in response to high-altitude exposure. Moreover, the relative abundance of specific microorganisms in both the pharyngeal and gut microbiota showed significant changes in response to variations in altitude, including certain opportunistic pathogens and species capable of producing functionally active metabolites. Some of these microorganisms exhibited significant differences between groups stratified by the presence or absence of self-reported respiratory or gastrointestinal symptoms during the high-altitude exposure. Together, these findings suggest a potential link between altitude-related microbiome shifts and host health status, which may reflect one of the pathways involved in acclimatization to hypoxic environments.

Our study revealed a significant decrease in alpha diversity, as measured by Shannon and Simpson indices, in the pharyngeal microbiota after 1 week of high-altitude acclimatization, whereas the gut microbiota showed a similar but nonsignificant downward trend shortly after arrival at high altitude. These findings align with limited existing evidence on pharyngeal microbiota, as Liu et al. (2021) reported that the alpha diversity of the oral microbiota decreased with increasing altitude.^15^ Reduced microbial diversity may compromise the host’s ability to resist pathogen colonization, potentially explaining the increased incidence of respiratory symptoms observed in our study and previous reports.^16^ In our data, pharyngeal ACE, Chao1, and Sobs indices exhibited a nonsignificant tendency to increase during high-altitude exposure. As ACE and Chao1 are richness-oriented estimators that are particularly sensitive to the number of rare taxa, whereas Shannon and Simpson jointly capture both richness and evenness, the combination of higher richness-oriented indices with lower Shannon and higher Simpson values suggests an expansion in total taxon richness accompanied by reduced evenness.^17,18^ This pattern is most likely driven by the disproportionate enrichment of a subset of taxa rather than a uniform increase across the whole community. This interpretation is supported by our taxon-level findings, in which several pharyngeal genera, such as *Streptococcus,* were markedly enriched at high altitude and may have dominated the community, thereby lowering overall evenness.

For the gut microbiome, our results are supported by recent epidemiologic evidence demonstrating decreased alpha diversity after two- to three-month high-altitude exposure.^7^ Evidence derived from animal experiments further corroborates that upon rapid exposure to high-altitude conditions, rats exhibited significantly lower values for the Shannon indices compared to control groups, while the Simpson index was markedly elevated.^19^ All gut alpha-diversity metrics remained essentially unchanged across visit phases, which may reflect a more buffered and ecologically stable community structure over the relatively short follow-up period.^20,21^

Hypoxic stress induced by high-altitude conditions may contribute to alterations in the diversity of pharyngeal and gut microbiota. The gut, as a central organ in stress response,^22^ is particularly susceptible to hypoxia-induced alterations, especially at elevations exceeding 5000 m.a.s.l.^23^ In the hypoxic environment, reduced intestinal peristalsis may impair the downward propulsion of bacteria, thereby affecting microbial colonization.^12^ Moreover, hypoxia upregulates the expression of hypoxia-inducible factor-1, which mediates the integrity of the epithelial barrier and affects epithelial ion transport function.^24^ These alterations may collectively influence the airway microenvironment and lead to changes in the composition of airway microbiota.^5^

Our study contributes novel evidence to the limited understanding of pharyngeal microbiota in high-altitude environments. The relative abundance of pathogenic or conditionally pathogenic bacteria in the pharyngeal microbiota showed an increasing tendency during high-altitude exposure. For example, *Streptococcus* exhibited a sustained increase in relative abundance throughout the high-altitude exposure. Similarly, a previous cross-sectional study on oral microbiota in Xizang residents (<3650 m) also identified *Streptococcus* as the dominant genus.^15^ *Solobacterium,* considered an indicator of oral hygiene,^25^ has been linked to diseases such as gastroesophageal reflux^26^ and colorectal cancer.^27^ Interestingly, in our study, the abundance of *Solobacterium* did not significantly differ between individuals with or without respiratory symptoms but was associated with gastrointestinal symptoms (*p* < 0.001). Potential interactions exist among microbial communities residing in distinct anatomical sites of the human body, so a comprehensive exploration is warranted. Notably, we found that *Peptostreptococcus* abundance remained stable during the high-altitude exposure but exhibited a significant increase after returning to the baseline level. This phenomenon may reflect environmental changes, such as differences in air pollution levels between Xizang and Beijing.^28^ Overall, the observed shifts in pharyngeal microbiota and their associations with host symptoms suggest potential implications for respiratory health and highlight the need for further research in well-controlled studies.

Concerning gut microbiota, we identified distinct changes in four genera, particularly, *Escherichia-Shigella* was significantly reduced by 15.03% after 1 week of high-altitude acclimatization. This finding is consistent with a previous study reporting decreased *Escherichia* and *Shigella* in high-altitude populations.^29^ The *Escherichia-Shigella* genus encompasses the two major gut commensals and has been associated with various diseases, including hypertension,^30^ heart failure,^31^ gastrointestinal inflammation,^30^ and kidney disease.^32^ Conversely, lower levels of *Escherichia-Shigella* have also been considered as a marker of improved gut health.^33^ We also noted that individuals self-reporting gastrointestinal symptoms during high-altitude exposure exhibited a lower relative abundance of this genus, suggesting it may be related to the impact of high-altitude environmental exposure on gastrointestinal health. In addition, our study found that the abundance of *Lachnoclostridium,* a key genus of *Lachnospiraceae*, was elevated during high-altitude exposure. *Lachnospiraceae* is associated with short-chain fatty acids (SCFAs) production and anti-inflammation,^15,34^ both of which may contribute to hypoxia acclimatization in the human gut.^7^ These taxonomic shifts may reflect microbiota-mediated mechanisms supporting host acclimatization to high-altitude environments.

In studies of chronic hypoxia, pronounced alterations in commensal microbial communities have also been documented. Previous work has reported that high-altitude residents show significant enrichment of 13 gut genera, including *Acidaminococcus*, *Actinomyces*, and *Prevotella*.^12^ This pattern is not entirely consistent with the taxonomic shifts observed in our study, which is likely attributable to differences in study design and microbiome profiling methodologies. From a functional perspective, however, comparative analyses indicate that the gut microbiota of Tibetan and Han populations differ substantially, and even among Han individuals, stool microbiomes from high-altitude residents exhibit a more energy-efficient community structure than those from low-altitude counterparts.^35^ Likewise, Andean highlanders are characterized by gut microbial profiles enriched in fiber-degrading and SCFA–producing taxa, suggesting an enhanced capacity to harvest energy from complex carbohydrates.^36^ In addition, functional predictions of oral microbiota have shown upregulation of amino acid and vitamin metabolism pathways in high-altitude populations.^15^ These lines of evidence indirectly support our results from acute hypoxia exposure, and suggest altitude-related restructuring of commensal microbiota is not only a biological response to hypoxic stress, but may also represent a potential adaptive mechanism, particularly through the modulation of energy metabolism.

In our study, gut taxa that differed across visit phases tended to covary with hypoxia-related indices in a manner consistent with greater hypoxemia, showing positive associations with HGB and inverse associations with SpO_2_, whereas pharyngeal genera generally exhibited parallel positive correlations with both HGB and SpO_2_. Although these correlations did not consistently reach statistical significance, the distinct covariation patterns between gut and pharyngeal communities suggest that altitude-responsive gut microorganisms may be more tightly linked to the host’s systemic hypoxic status. By contrast, pharyngeal microbiota, which are directly exposed to inhaled air and airway conditions, are likely influenced by both the internal host milieu and the external environment, potentially reflecting a composite response to hypoxia and local environmental factors.

Our study has certain advantages. First, by employing a longitudinal quasi-experimental design, we were able to track the dynamic changes in the microbial community throughout the acute physiological acclimatization process, as lowland participants rapidly ascended to high-altitude regions. Repeated sampling and within-subject comparisons enabled better control of interindividual heterogeneity in microbiota detection. Second, our study concurrently examined commensal microbiota from multiple body sites, contributing to a more comprehensive understanding of how high-altitude exposure affects microbial communities across anatomical niches. The consistent trend of reduced microbial diversity across sites strengthens our confidence in these observations.

Some limitations should also be acknowledged. First, the use of 16S rRNA gene sequencing only permitted taxonomic resolution at approximately the genus level, which constrained our ability to capture strain-level heterogeneity, infer specific functional capacities, and directly link microbial shifts to detailed mechanistic pathways of high-altitude acclimatization. Second, as a quasi-experimental study, we cannot exclude the possibility of residual confounding from unmeasured factors. In particular, we did not rigorously control or quantitatively assess individual dietary intake, which may have introduced residual confounding and reduced our power to detect more subtle microbiome changes, especially for the gut microbiota. However, during the high-altitude stay, all participants were provided with centralized meals broadly comparable to typical lowland diets. Together with the repeated-measures design in which each participant served as his or her own control, this relatively stable habit and similar food supply pattern are likely to have attenuated the impact of diet on our main findings. Moreover, the entire follow-up at high altitude was completed within a period of less than one month. Previous evidence shows that even under controlled dietary interventions, detectable community-level changes typically require several weeks or even months to emerge.^20,21^ Nevertheless, we acknowledge that detailed information on diet and other potential confounders was limited in the present study, and our results should be further validated in future work with more comprehensive confounder assessment and, ideally, controlled or standardized protocols to more clearly disentangle the roles of diet, other lifestyle factors, and hypoxia exposure.

## Conclusions

In summary, this study provides novel evidence for the critical role of the human microbiome in high-altitude acclimatization. By dynamically tracking changes in the pharyngeal and gut microbiota during the acclimatization process, we found that the pharyngeal microbiome exhibited more pronounced alterations, characterized by reduced alpha diversity and increased abundance of pathogens and potentially pathogenic taxa. These changes may contribute to respiratory symptoms and infections commonly reported in high-altitude regions. Furthermore, the observed decline in gut microbial diversity, particularly the reduction of *Escherichia-Shigella*, may negatively impact gastrointestinal health and be associated with gastrointestinal symptoms. Our results highlight the importance of considering the human microbiome as an integral component of acclimatization to extreme environments. Future research should aim to elucidate the mechanistic pathways linking microbiome changes with health outcomes and to inform targeted interventions to support human health and performance in extreme environments.

## Supplementary Material



## Data Availability

The raw sequence data reported in this paper have been deposited in the Genome Sequence Archive in the National Genomics Data Center (HRA011528; HRA011529), which are publicly accessible at https://ngdc.cncb.ac.cn/gsa.

## Notes

Detailed descriptions of oxygen supplementation and antibiotic use (Tables S1 and S2); descriptive statistics and analytical results for pharyngeal and gut microbiota alpha and beta diversity (Tables S3 and S4, Figure S2); changes in hypoxia-related physiological indicators across clinical visits (Figure S1) and results of differential microbiota analyses (Figure S3, Tables S5 and S6); distributions of differential taxa grouped by self-reported respiratory and gastrointestinal symptoms (Figure S4); Spearman correlation analyses between differential taxa and hypoxia-related physiological indicators (Figure S5); and sensitivity analyses accounting for potential confounders (Figures S6–S9) (PDF)
