# Supplementary material for: Short-Term High-Altitude Exposure Alters Pharyngeal and Gut Microbiome
Source: Environ Health (Wash). 2026 Jan 14;4(5):1002–11. doi: 10.1021/envhealth.5c00585 (PMC13185073; doi:10.1021/envhealth.5c00585)
Supplement: Supplementary file 1 [file eh5c00585_si_001.pdf]

Supporting Information

Short-Term High-Altitude Exposure Alters Pharyngeal and Gut Microbiome

Yi Zhang[1,2#], Xin Meng[1,2#], Jicheng Gong[1,2,3*], Yifan Xu[1,2,3], Qiaoyi Hua[1,2], Wu Chen[1,2], Teng Wang[1,2], Xudong He[4], Ruiwei Xu[1,2], Yingfeng Gao[1,2], Chunxiang Ye[1,2,3], Cencen Wu[2,5], Yuanyuan Fan[2,5], Lingyun Zu[2,5*], Tong Zhu[1,2,3*]

[1]MEEKL-AERM, College of Environmental Sciences and Engineering, Institute of Tibetan Plateau, and Center for Environment and Health, Peking University, Beijing 100871, China.

[2] Institute of Tibetan Plateau, Peking University, Beijing 100871, China

[3] TUWAS-AEEEH, Xizang University, Lhasa, Xizang 850000, China.

[4] Medical College of Xizang University, Lhasa, Xizang 850000, China.

[5]Department of Cardiology and Institute of Vascular Medicine, Peking University Third Hospital; State Key Laboratory of Vascular Homeostasis and Remodeling, Peking University; NHC Key Laboratory of Cardiovascular Molecular Biology and Regulatory Peptides, Peking University; Beijing Key Laboratory of Cardiovascular Receptors Research. Beijing 100191, China.

[#] Ms Zhang and Dr Meng contributed equally.

[*]Correspondence to:

Jicheng Gong, PhD, MEEKL-AERM, College of Environmental Sciences and Engineering, Institute of Tibetan Plateau, and Center for Environment and Health, Peking University, Beijing 100871, China; TUWAS-AEEEH, Xizang University, Lhasa, Xizang 850000, China.

E-mail: jchgong@pku.edu.cn

Tong Zhu, ScD, MEEKL-AERM, College of Environmental Sciences and Engineering, Institute of Tibetan Plateau, and Center for Environment and Health, Peking University,

Beijing 100871, China; TUWAS-AEEEH, Xizang University, Lhasa, Xizang 850000, China. E-mail: tzhu@pku.edu.cn

Lingyun Zu, PhD, Department of Cardiology and Institute of Vascular Medicine, Peking University Third Hospital; State Key Laboratory of Vascular Homeostasis and Remodeling, Peking University; NHC Key Laboratory of Cardiovascular Molecular Biology and Regulatory Peptides, Peking University; Beijing Key Laboratory of Cardiovascular Receptors Research; Institute of Tibetan Plateau, Peking University. Beijing 100191, China. E-mail: dr_zly@126.com

Supplementary material

Table S1. Oxygen Supplementation Among Study Participants During The Study Period.......4

Table S2. Antibiotic Use Among Study Participants During The Study Period........................4

Table S3. Statistics Of Alpha Diversity In Pharyngeal And Gut Microbes. 5

Figure S1. Percent Change (%) In Hemoglobin (Hgb) And Peripheral Capillary Oxygen Saturation (Spo_2) Across Four Visits...6

Figure S2. Principal Coordinate Analysis Of Gut Microbiota (A) And Pharyngeal Microbiota (B) Based On Bray-Curtis Distances Of The Microbial Community At Different Clinic Visits. Colors Indicate Health Visits. Dots Represent Different Samples At Four Clinic Visits. Permutation Multiple Analysis Of Variance Is Used To Determine Significance. Adjusted R^2 Explains The Adjusted Effect Size. 7

Table S4. Results Of Permutation Multiple Analysis Of Variance Analysis Of Gut And Pharyngeal Microbes At Different Clinic Visits. 8

Table S5 Results Of Lefse Analysis Of Pharyngeal Differential Microbes. 9

Table S6 Results Of Lefse Analysis Of Gut Differential Microbes. 10

Figure S3. Relative Abundance In The Differential Gut And Pharyngeal Microbiota At Four Visits. 11

Figure S4. Relative Abundance Of Different Gut And Pharyngeal Microbiota In Populations With/Without Gastrointestinal (A) Or Respiratory Symptoms (B). 12

Figure S5. Spearman Correlations Between Differential Pharyngeal And Gut Microbial Genera And Hemoglobin (Hgb) And Peripheral Blood Oxygen Saturation (Spo_2). 13

Figure S6. Differences In Alpha Diversity Indices Of Pharyngeal And Gut Microbiota Across Four Visits After Exclusion Of Antibiotic-Exposed Visits. 14

Figure S7. Differences In Alpha Diversity Indices Of Pharyngeal And Gut Microbiota Across Four Visits After Further Adjusted For Oxygen Supplementation. 15

Figure S8. Differences In The Differential Gut And Pharyngeal Microbiota At Four Visits After Exclusion Of Antibiotic-Exposed Visits. 16

Figure S9. Differences In The Differential Gut And Pharyngeal Microbiota At Four Visits After Further Adjusted For Oxygen Supplementation..17

Table S1. Oxygen supplementation among study participants during the study period.

Participant ID	Frequency	Duration per session (hour)	Type	Visit	Time before clinical visit (day)
7	1	1	Canned oxygen	Visit 2	5
8	1	1	Diffuse oxygen	Visit 2	14
19	1	1-2	Diffuse oxygen	Visit 2	5
3	1	0.5	Diffuse oxygen	Visit 2	14
1	2	0.5	Diffuse oxygen	Visit 2	14,13
2	1	3	Diffuse oxygen	Visit 2	2
9	1	0.5	Diffuse oxygen	Visit 2	14
20	1	2	Diffuse oxygen	Visit 3	2
15	1	0.5	Diffuse oxygen	Visit 3	4
14	1	1	Diffuse oxygen	Visit 4	24

Table S2. Antibiotic use among study participants during the study period

Participant ID	Frequency	Visit	Time before clinical visit (day)
2	1	Visit 2	6
17	1	Visit 4	10

Table S3. Statistics of alpha diversity in pharyngeal and gut microbes.

	**Pharyngeal microbiota**	**Gut microbiota**
**Sobs**	90.8 ± 32.1	37.5 ± 9.5
**ACE**	96.2 ± 37.3	38.5 ± 9.8
**Chao 1**	96.2 ± 34.8	37.9 ± 9.7
**Shannon**	2.78 ± 0.31	1.61 ± 0.41
**Simpson**	0.11 ± 0.06	0.32 ± 0.14

Data are represented as mean ± standard deviation.

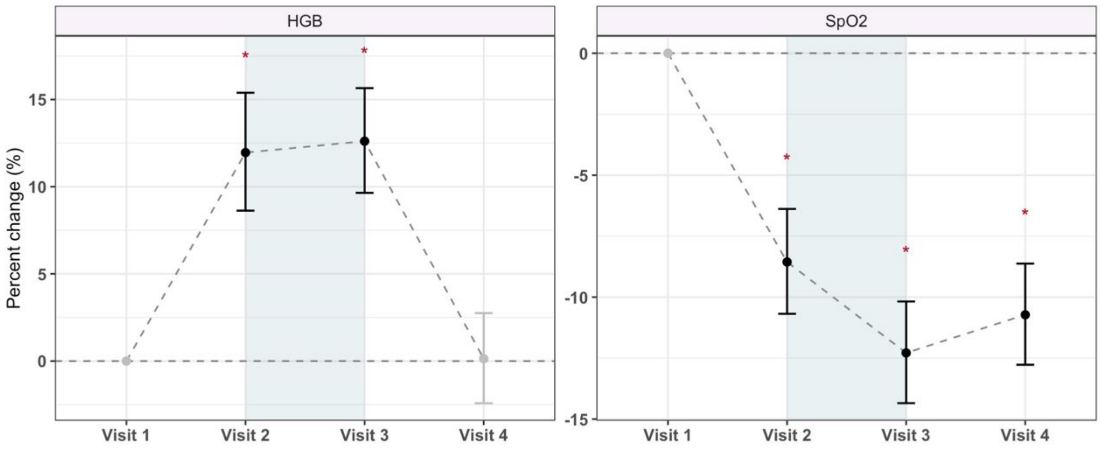

Figure S1. Percent change (%) in hemoglobin (HGB) and peripheral capillary oxygen saturation (SpO_2) across four visits.

Results were obtained from linear mixed-effects models with random intercepts of participants and adjusted for age, gender, smoking status, and body mass index (BMI). All estimates are reported as percent differences with 95% confidence intervals compared with the baseline (Visit 1), and no intersection with the horizontal dotted line indicates significant differences (in black). Differences with $FDR_{B\text{-}H} < 0.05$ are marked with asterisks. Blue shading indicates visits conducted at the high-altitude region.

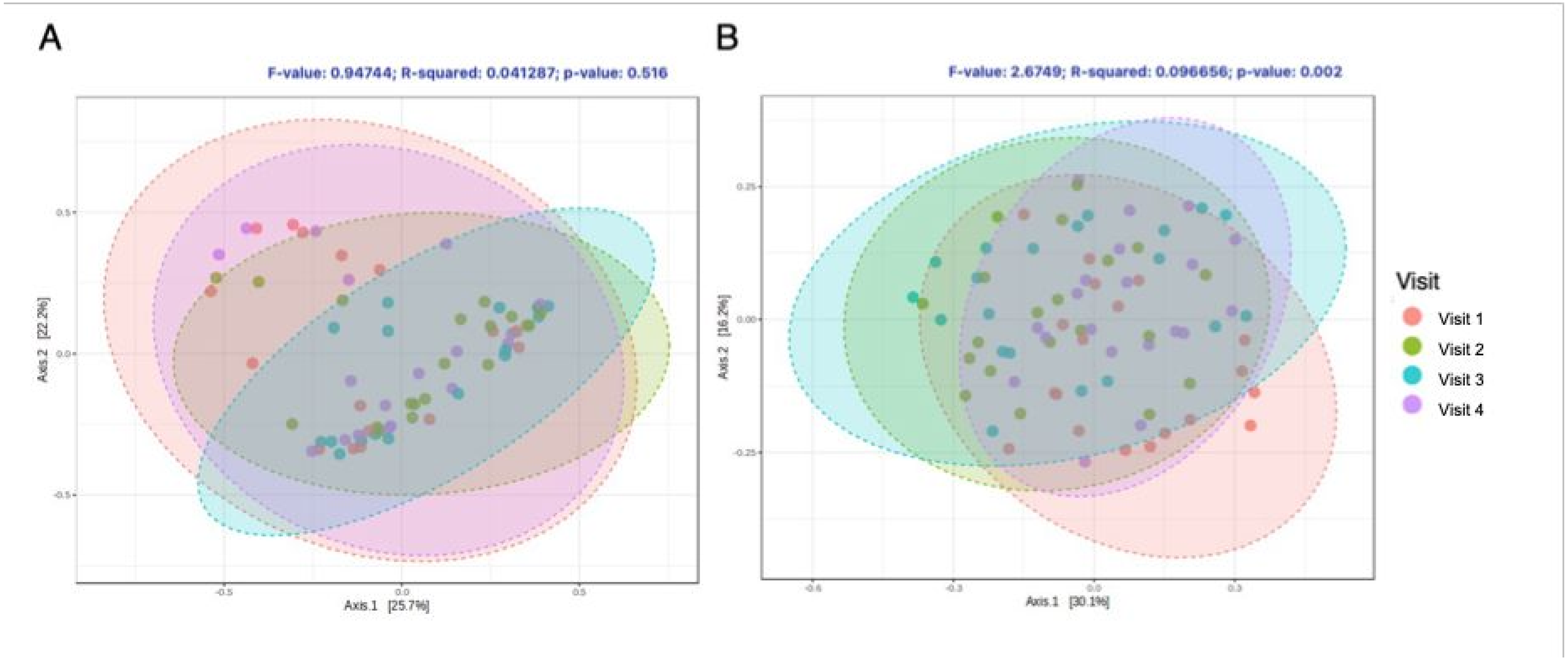

Figure S2. Principal coordinate analysis of gut microbiota (A) and pharyngeal microbiota (B) based on Bray-Curtis distances of the microbial community at different clinic visits. Colors indicate health visits. Dots represent different samples at four clinic visits. Permutation multiple analysis of variance is used to determine significance. Adjusted R^2 explains the adjusted effect size.

Table S4. Results of permutation multiple analysis of variance analysis of gut and pharyngeal microbes at different clinic visits.

	**Gut microbiota**				**Pharyngeal microbiota**			
	**F-value**	**R^2**	***p* value**	**FDR**	**F-value**	**R^2**	***p* value**	**FDR**
**visit 3 vs visit 1**	1.271	0.039	0.198	0.843	3.478	0.086	0.003	0.009
**visit 3 vs visit 4**	0.896	0.027	0.562	0.843	4.325	0.105	0.002	0.009
**visit 3 vs visit 2**	0.407	0.012	0.994	0.994	2.062	0.053	0.030	0.036
**visit 1 vs visit 4**	0.438	0.013	0.968	0.994	1.074	0.027	0.358	0.358
**visit 1 vs visit 2**	1.003	0.029	0.398	0.843	2.359	0.058	0.021	0.032
**visit 4 vs visit 2**	0.943	0.026	0.487	0.843	3.131	0.076	0.010	0.020

Table S5 Results of LEfSe analysis of pharyngeal differential microbes.

	p value	FDR	Visit 1	Visit 2	Visit 3	Visit 4	LDA score
Streptococcus	<0.001	0.010	1253500	1600600	2436000	1332000	5.77
Acinetobacter	0.001	0.029	477970	81967	2464	7456	5.38
Haemophilus	0.002	0.029	460900	1048700	927750	504190	5.47
Alloprevotella	0.002	0.029	403230	218100	152050	411040	5.11
Bergeyella	0.006	0.058	17542	54084	39200	10505	4.34
f_Actinomycetaceae_g_F0332	0.006	0.058	1177	2895	8110	1370	3.54
Porphyromonas	0.017	0.118	149100	342720	187680	139390	5.01
Solobacterium	0.017	0.118	163330	72899	67779	139120	4.68
Delftia	0.024	0.146	9974	3716	4040	959	3.65
Peptostreptococcus	0.029	0.161	134730	104420	100290	275360	4.94
Atopobium	0.043	0.217	146080	74067	77155	172820	4.69

Table S6 Results of LEfSe analysis of gut differential microbes.

	p value	FDR	Visit 1	Visit 2	Visit 3	Visit 4	LDA score
Escherichia-Shigella	0.003	0.190	2291600	817040	365320	1907100	5.98
Clostridium_sensu_stricto_1	0.023	0.556	262020	81247	43212	16475	5.09
Lachnoclostridium	0.030	0.556	84470	30459	60373	239150	5.02
Novosphingobium	0.050	0.658	6010	84975	27097	15824	4.60

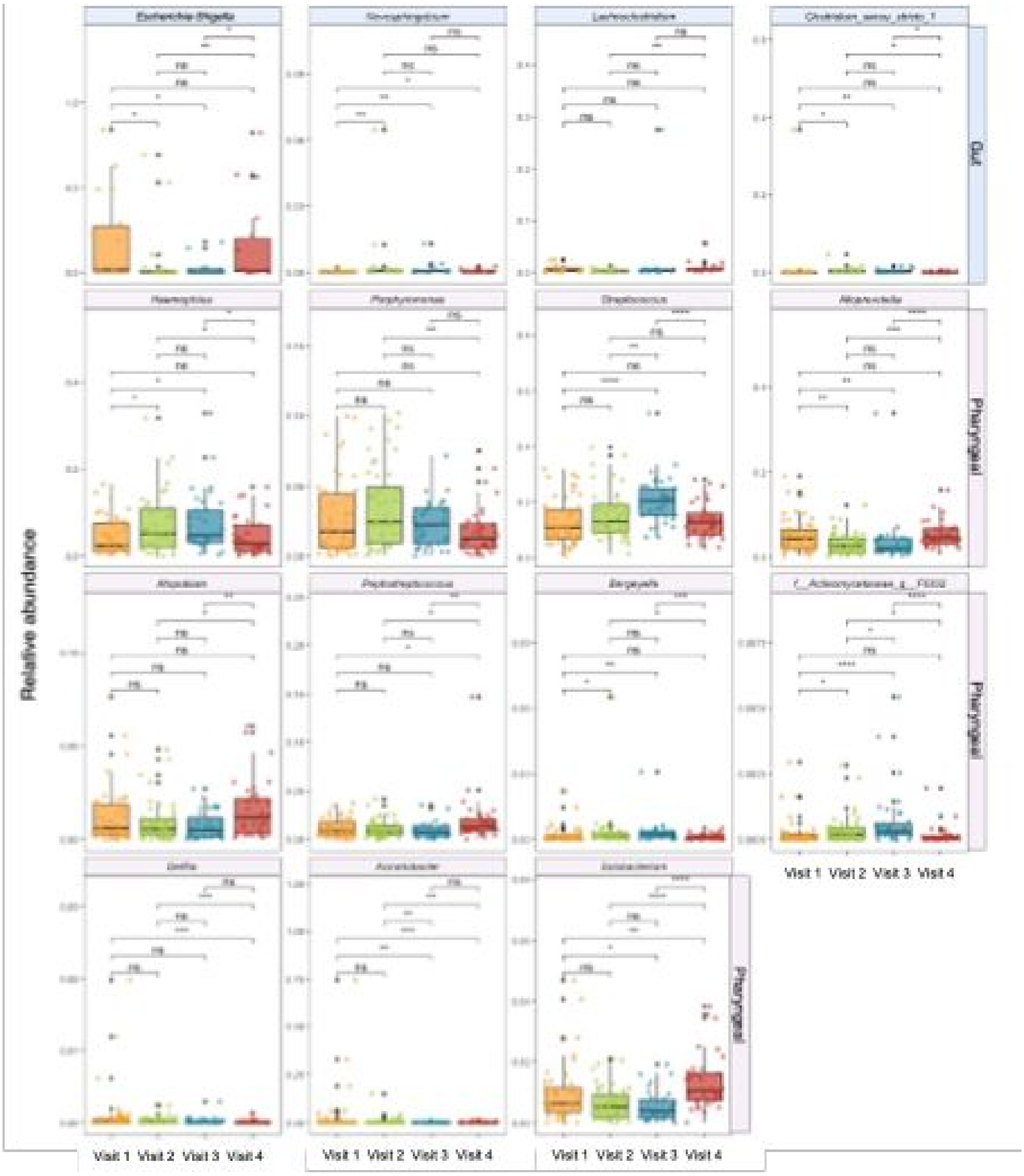

Figure S3. Relative abundance in the differential gut and pharyngeal microbiota at four visits.

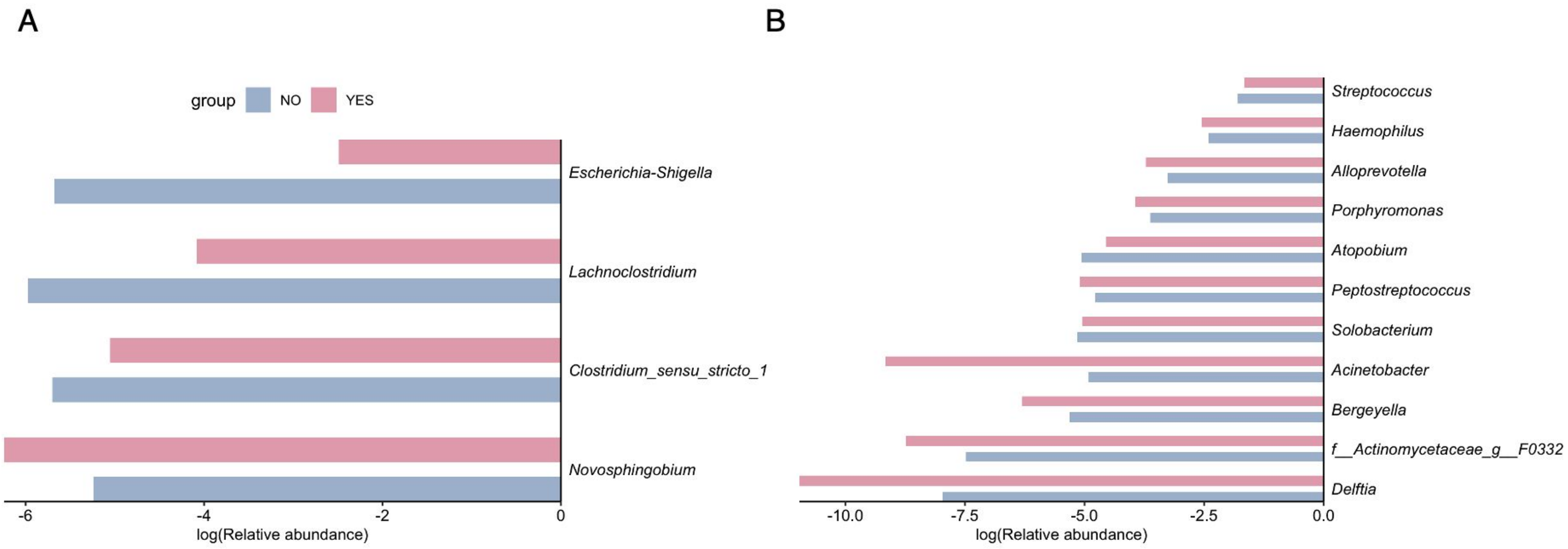

Figure S4. Relative abundance of different gut and pharyngeal microbiota in populations with/without gastrointestinal (A) or respiratory symptoms (B).

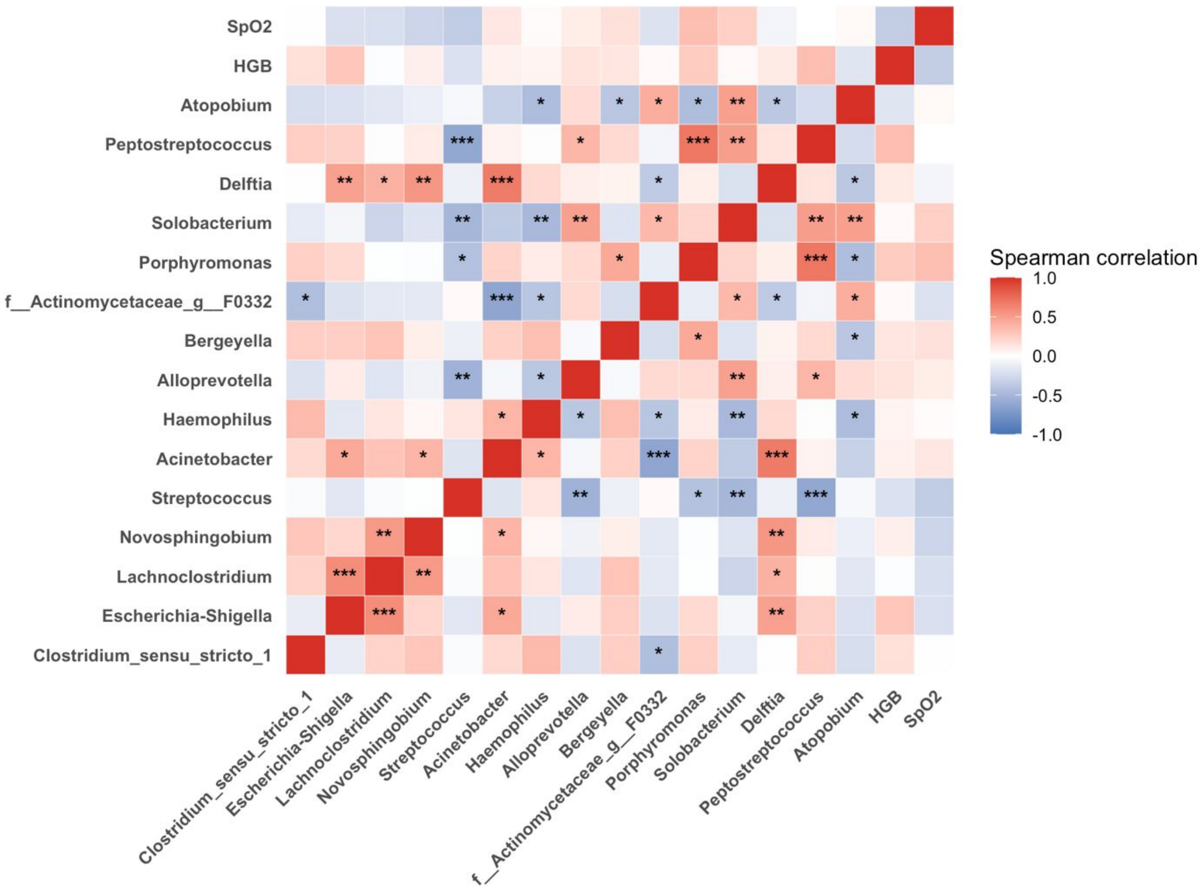

Figure S5. Spearman correlations between differential pharyngeal and gut microbial genera and hemoglobin (HGB) and peripheral blood oxygen saturation (SpO_2).

Note: **p* < 0.05, ***p* < 0.01, ****p* < 0.001.

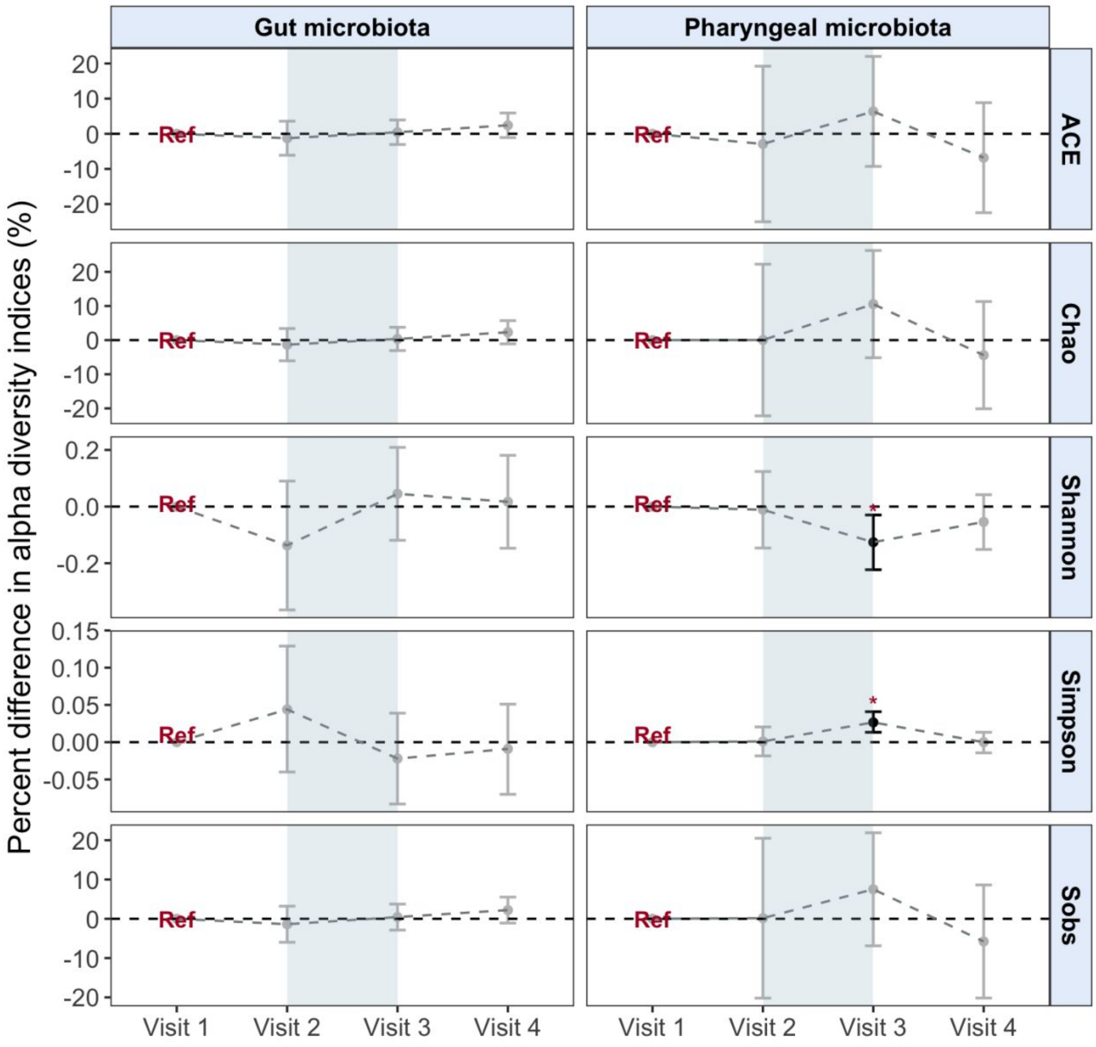

Figure S6. Differences in alpha diversity indices of pharyngeal and gut microbiota across four visits after exclusion of antibiotic-exposed visits.

Results were obtained from linear mixed-effects models with random intercepts of participants and adjusted for age, gender, smoking status, and body mass index (BMI). All estimates are reported as percent differences with 95% confidence intervals compared with the baseline (Visit 1), and no intersection with the horizontal dotted line indicates significant differences (in black). Differences with $FDR_{B\text{-}H} < 0.05$ are marked with asterisks. Blue shading indicates visits conducted at the high-altitude region.

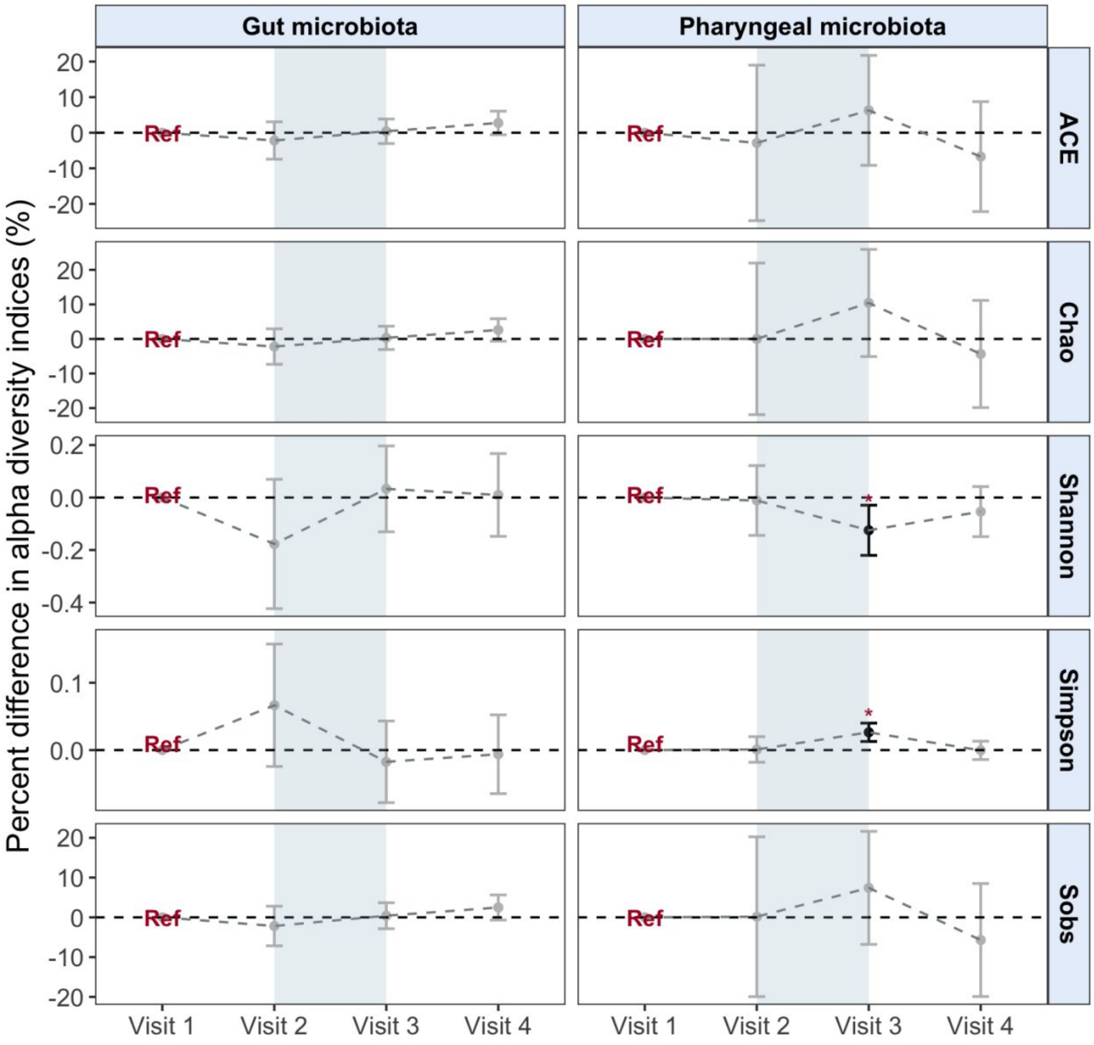

Figure S7. Differences in alpha diversity indices of pharyngeal and gut microbiota across four visits after further adjusted for oxygen supplementation.

Results were obtained from linear mixed-effects models with random intercepts of participants and adjusted for age, gender, smoking status, body mass index (BMI), antibiotic use, and oxygen supplementation. All estimates are reported as percent differences with 95% confidence intervals compared with the baseline (Visit 1), and no intersection with the horizontal dotted line indicates significant differences (in black). Differences with $FDR_{B\text{-}H} < 0.05$ are marked with asterisks. Blue shading indicates visits conducted at the high-altitude region.

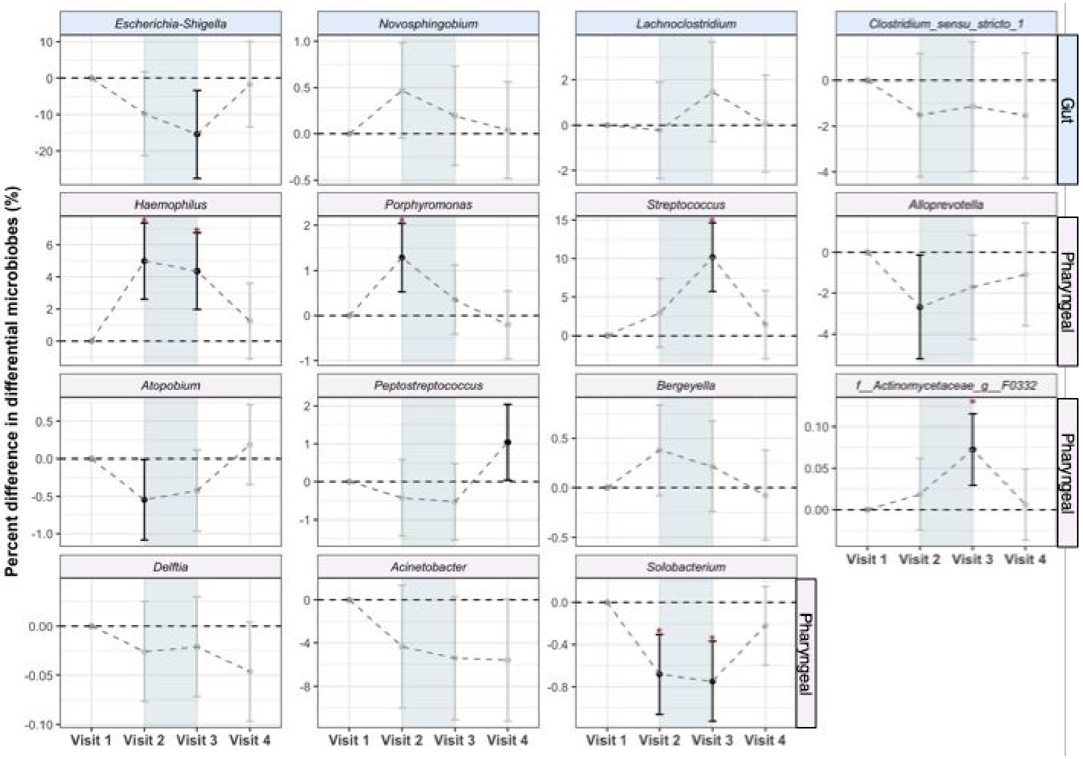

Figure S8. Differences in the differential gut and pharyngeal microbiota at four visits after exclusion of antibiotic-exposed visits.

Results were obtained from linear mixed-effects models with random intercepts of participants and adjusted for age, gender, smoking status, and body mass index (BMI). All estimates are reported as percent differences with 95% confidence intervals compared with the baseline visit (Visit 1), and no intersection with the horizontal black dotted line indicates significant differences (in black). Differences with $FDR_{B\text{-}H} < 0.05$ are marked with asterisks. Blue shading indicates visits conducted at the high-altitude region.

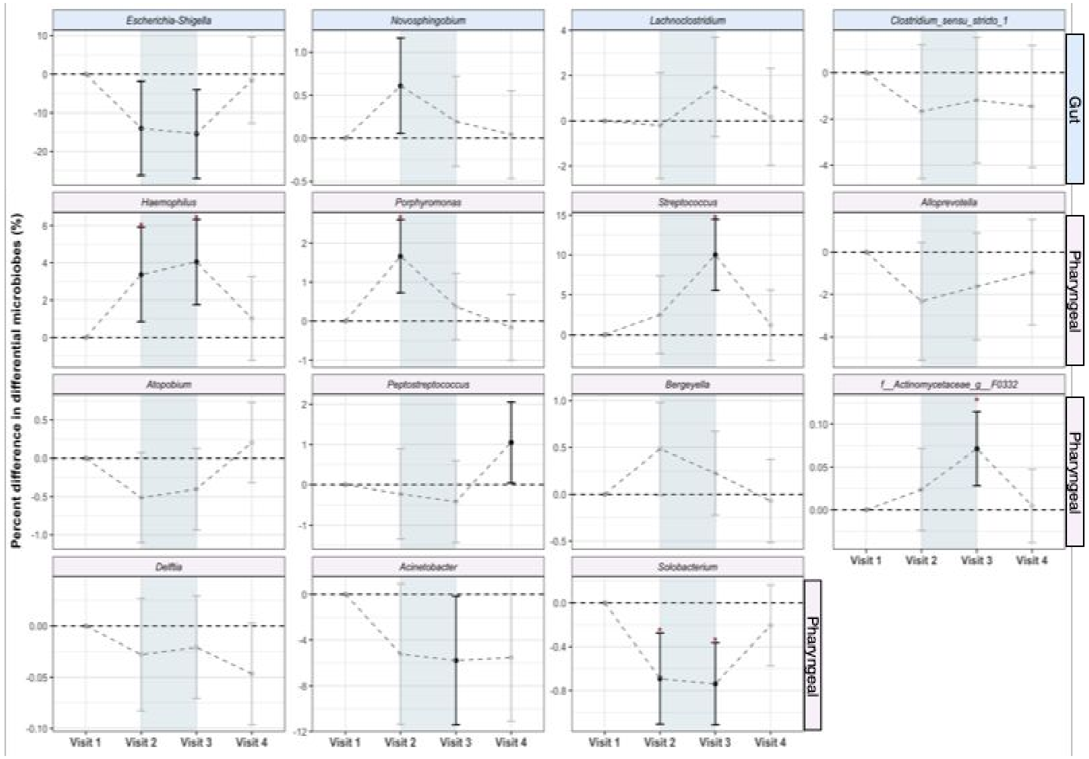

Figure S9. Differences in the differential gut and pharyngeal microbiota at four visits after further adjusted for oxygen supplementation.

Results were obtained from linear mixed-effects models with random intercepts of participants and adjusted for age, gender, smoking status, body mass index (BMI), antibiotic use, and oxygen supplementation. All estimates are reported as percent differences with 95% confidence intervals compared with the baseline visit (Visit 1), and no intersection with the horizontal black dotted line indicates significant differences (in black). Differences with $FDR_{B\text{-}H} < 0.05$ are marked with asterisks. Blue shading indicates visits conducted at the high-altitude region.
